# Oxford on Its Mettle

**Published:** 1891-05-09

**Authors:** 


### The Hospital

A Weekly Journal of

**Science, Medicine, Nursing, and Philanthropy.**

*For Hospitals, Asylums, Infirmaries, Dispensaries, Medical Practitioners, Students, Nurses, and the Charitable Public.*

Vol. X.—No. 241.] [May 9, 1891.

### Oxford on its Mettle.

The Master of University College and twenty-eight of his fellow citizens met last week to consider their duty to the Radcliffe Infirmary. The committee consisted of more than a dozen clergymen, three or four doctors, a soldier, and a sprinkling of the ordinary laity. Lord Clarendon we know had a poor opinion of the business capacity of the clergy; and Macaulay quoted Clarendon's famous dictum with evident enjoyment and approbation. We shall not be so uncivil as to reproduce here either Clarendon's opinion or Macaulay's comments thereupon. Even if we did, it would only be to show how very much the clergy, at any rate at Oxford, have improved in business capacity since Clarendon's time. For it is evident to any one who reads the report of the discussion which was initiated by Dr. Bright that the clergy had the best of the running. Dr. Bright himself, and the Rev. C. H. J. Fletcher made speeches of a business-like character and worthy of the occasion.

It is true that Dr. Bright gently rallied The Hospital, and even indulged in a little acerbity of tone towards our Special Commissioner. That we were quite prepared for. The Hospital has always believed in "give and take." When we decided that it was necessary to come down upon Dr. Bright's toes, we felt quite sure that he would retort by giving us a sound cuff on the ear. This is quite English, and altogether as it should be.

But the affairs of the Oxford Infirmary still stand between the two opposing parties; and it seems to us that the best course to pursue is to fall to a practical and friendly consideration of the difficulties of the moment. What are those difficulties? We will not answer this question by repeating the statements of our Commissioner; we will, on the contrary, take the admissions of Dr. Bright himself, and of the other members of the Infirmary Committee. But we will not use those admissions with the poor object of vindicating our Commissioner. What we wish to do is to persuade the authorities to so deal with their hospital as to make it all that it should be as a place of healing for the sick poor, and worthy, in every sense, of England's greatest and most honoured University. It is not possible to believe that medical and sanitary writers in London should be more jealous of Oxford's honour than Oxford is herself; and yet we cannot but feel that, compared with Cambridge and Edinburgh, or even with Glasgow and Dublin, Oxford is to the last degree unworthily represented by its hospital and its medical school.

The Radcliffe Infirmary, to a hospital expert, looks from the outside gloomy and depressing. Is that the fact? Dr. Bright said at the meeting of the Committee, according to the *Oxford Chronicle's* report, that "the impression raised by the appearance on an ordinary person used to seeing hospitals when he first entered it was that it was gloomy. The whole place looked heavy and gloomy, and he could not help thinking that it was a real impression, and that must be their feeling when they first entered it." There need be then no further discussion about the outside appearance of the hospital. The place, by Dr. Bright's own admission, is "heavy and gloomy." But if it is gloomy it is melancholy, and we know it to be old. Will anybody in these days contend that a hospital which is admitted by its own friends to be heavy, gloomy, melancholy, and antiquated, is the kind of place to which persons suffering from severe and depressing illness should be sent to recover their health?

But it is plain that Dr. Bright and his colleagues knew very well that their hospital was in a highly unsatisfactory condition long before our Commissioner appeared on the scene. For what does Dr. Bright say on this point? He reminds his fellow-managers that when he was first appointed Treasurer seven years ago "they entered upon a plan for the entire reconstruction of a large portion of the hospital. Two plans were introduced, but both of them rested on the feeling that the old buildings were insufficient for the perfection of the hospital." But is not this one fact, that the managers themselves, seven years ago, thought it absolutely necessary to reconstruct a large portion of the hospital, an argument which is a thousand times more condemnatory of the old buildings than any statement put forward by our Commissioner? Why was it then thought necessary to entirely reconstruct a large portion of the building? And why has the reconstruction not been carried out? That it was not merely the gloom and melancholy of the outside of the hospital which suggested reconstruction we may be quite sure; for practical Englishmen could not have been persuaded to contemplate the spending of ten thousand pounds on mere appearances. It is evident that the managers, who were perfectly familiar with every detail of the structure and possibilities of their building at that time felt that it was very far from being an ideally suitable place for the care and cure of the sick. Actions notoriously speak louder than words; and if Dr. Bright and his supporters were to argue until doomsday they could not diminish by a hair's breadth the practical significance of the fact that they had already determined, without any outside criticism at all, to reconstruct a large portion of their old building.

The Rev. C. J. H. Fletcher, a practical observer and a convincing speaker, put all our Commissioner's criticisms into a couple of sentences, and confirmed their accuracy. These are his words as reported in the *Oxford Chronicle*: "The London critic had put his finger on the weak points in this institution. Their main building, he told them, was antiquated, its three wards were unsatisfactory, and the accommodation it provided was insufficient. The Committee did not deny, they admitted these defects. In their last year's report they said that the wards in the old buildings were old-fashioned and could not be considered satisfactory, that further accomodation for the nurses was much wanted, and that the kitchen offices required re-arrangement and improvement. They had a plan in view for remedying these imperfections." Mr. Fletcher reminded his confrères that the building was one hundred and fifty years old, and that more than a century ago no less a person than John Howard, the philanthropist, had condemned it, after inspection, in no measured terms. He contended that the building should be pulled down and replaced "by a structure of greater beauty, and more adapted to the present state of hospital administration." "Shall we," asked Mr. Fletcher, "hand down to the twentieth century this antiquated block, which did not satisfy the requirements of the eighteenth?"

The medical staff seem to have kept themselves a little in the background since the publication of their early protest. But Mr. Winkfield, F.R.C.S., senior surgeon to the hospital, could not withhold his opinion of its internal condition when pointedly appealed to. Speaking of the three old wards which have been so much discussed, he said that "of course they," that is the surgeons, "did not reckon them so good as the more airy wards out in the garden, and they would be glad to have the opportunity of transferring the patients from the wards in the house to the more modern and more airy wards in the garden. The old wards had the disadvantage of being in the general building, with the various odours and currents coming from the kitchen and offices downstairs, which was not good for the patients. . . No doubt if the funds could be obtained the plan which Mr. Fletcher had hinted at of pulling down the old building and re-building it would be the better one."

Will Dr. Bright, after these admissions by members of the Committee, contend that The Hospital and its Special Commissioner have gone beyond the lines of plain duty in calling the attention of the Oxford managers to the state of their Infirmary? Much more properly might he argue that The Hospital had no *raison d'être* for its existence if it failed to call attention to such institutions, and to do it with such pointedness and energy as to ensure immediate consideration. It is as clear as facts can make it that the old Radcliffe Infirmary ought to be pulled down. Dr. Bright and his colleagues know this quite as well as we do, and all Oxford knows it. Derby is pulling down its old building, and has raised within a fortnight £40,000 of the £70,000 required for its new buildings. Oxford is relatively a much richer county than Derby. Its gross rental is half that of Derbyshire, whilst its population is only two-fifths. Oxford must build itself a new hospital. The honour of the county, of the city, and of the University alike demands it. We hope that, notwithstanding any little sensitiveness which our criticisms may have aroused, we shall not be forbidden the honour of a share in the erection of the new building.

